# Addressing psychosomatic symptom distress with mindfulness-based cognitive therapy in somatic symptom disorder: mediating effects of self-compassion and alexithymia

**DOI:** 10.3389/fpsyt.2024.1289872

**Published:** 2024-02-07

**Authors:** Lianrong Xu, Jihong Shi, Chengwen Li

**Affiliations:** ^1^Department of Pain Management, Jinxiang Hospital Affiliated to Jining Medical University, Jining, China; ^2^Department of Consultation Psychology, Jining First People's Hospital, Jining, China; ^3^Department of Anesthesiology, Beijing Friendship Hospital, Capital Medical University, Beijing, China

**Keywords:** somatic symptom disorder, mindfulness-based cognitive therapy, self-compassion, alexithymia, psychosomatic distress

## Abstract

**Objective:**

This study explored the association between self-compassion, alexithymia, and psychosomatic symptom distress in a clinical sample of somatic symptom disorder (SSD) patients participating in a mindfulness-based cognitive therapy (MBCT) program.

**Methods:**

One hundred sixteen SSD patients who had participated in an MBCT program and completed ≥4 intervention sessions were included in a retrospective study (76.7% women, mean age = 40.0, SD = 9.5). Psychometric measures of psychosomatic symptom distress [Brief Symptom Inventory-18 Global Severity Index (BSI-GSI)], self-compassion [Self-Compassion Scale (SCS)], and alexithymia [Toronto Alexithymia Scale (TAS)] were collected upon admission to the MBCT program and at 6-month follow-up following treatment inclusion.

**Results:**

Serial mediation analysis (MBCT→ΔSCS→ΔTAS→ΔBSI-GSI) suggested that changes in both self-compassion and alexithymia had significant indirect effects on improvement in psychosomatic distress [ΔSCS β = −1.810, 95% bootstrap CI (−2.488, −1.160); ΔTAS β = −1.615, bootstrap 95% CI (−2.413, −0.896); ΔSCS→ΔTAS β = −0.621, bootstrap CI (−1.032, −0.315)]. Furthermore, a *post-hoc* analysis with a reverse sequence (MBCT→ΔTAS→ΔSCS→ΔBSI-GSI) revealed that reduction in alexithymia improved psychosomatic distress and that an increase in self-compassion was a subsequent outcome of alleviation of alexithymia [ΔTAS β = −2.235, bootstrap 95% CI (−3.305, −1.270); ΔSCS β = 0.013, 95% bootstrap CI (−0.600, 0.682); ΔTAS→ΔSCS β = −1.823, bootstrap CI (−2.770, −1.047)].

**Conclusion:**

Both alleviation of alexithymia and improvement in self-compassion play a mediating role in the reduction of psychosomatic distress in SSD patients following an MBCT program. Improvement in self-compassion might be a subsequent outcome of MBCT-related alleviation of alexithymia.

## Introduction

Somatic symptom disorder (SSD), a new term introduced to replace the diagnostic label of somatoform disorder (SFD) in the Diagnostic and Statistical Manual of Mental Disorders, 5th edition (DSM-V), is characterized by prominent physical symptoms that are associated with marked distress and impairment, including excessive thoughts, feelings, and behaviors relating to the physical disturbance (1). It is well established that cognitive and emotional factors, including dysfunctional beliefs, are central to the onset, aggravation, and maintenance of SSD (2–5). Impairment of cognitive–emotional regulation is a hallmark of SSD (6). Cognitive behavioral therapy (CBT) is considered to be well established as a treatment for SSD, but at best, it produces moderate improvement (7). A new generation of CBT has been developed by integrating this approach with mindfulness skills. Forms of this new-generation CBT, such as mindfulness-based cognitive therapy (MBCT), may be promising in this context. MBCT is a specific form of mindfulness-based intervention (MBI), based on the mindfulness-based stress reduction (MBSR) program developed by Kabat-Zinn (8). Accumulating evidence has demonstrated the efficacy of both CBT and MBIs in mitigating symptom severity, psychological distress, and disability in SSD (9–11). Mindfulness training and cognitive restructuring as strategies for emotion regulation are core components of MBCT. However, the specific active components and mechanisms of change in MBCT remain less clear.

Systematic reviews have emphasized the importance of identifying components that mediate MBCT outcomes (12, 13). It is widely accepted that changes in mindfulness mediate treatment outcomes (14–16). It has been argued that mindfulness training is one way to promote self-compassion in the context of overall wellbeing (17–20). MBCT sometimes implicitly communicates components of self-compassion, unlike compassion-focused therapy, which explicitly teaches self-compassion skills (21). Multiple studies have demonstrated an association between improved self-compassion and reduced negative emotion (16, 17, 22–26). Self-compassion is regarded as a mediating or moderating variable in MBIs (19, 27). Nonetheless, a systematic review failed to identify this relationship (28).

An additional trait that may influence the outcomes of MBCT is alexithymia, a deficit in emotional clarity, which involves difficulties with monitoring, identifying, and describing emotions. Alexithymia is generally acknowledged as a risk factor for both somatic and mental pathologies (29), which can interfere with health perception and emotion regulation, resulting in increased negative affect and lower health-related quality of life. Mindfulness meditation has the potential to counteract alexithymia to an extent by enhancing open curiosity and attentiveness to inner experiences and increasing familiarity with the thoughts or feelings appearing in the body (30). Systematic reviews have shown that MBIs can improve emotional clarity (31–33). Nevertheless, Butler et al. have denied any effect of emotional clarity on treatment outcomes (33).

Self-compassion and alexithymia may interact over the course of MBCT. Self-criticism is an internal process contrary to self-compassion, and alexithymia has been found to be associated with it (30). The primary objectives of the present study were twofold. First, the present study aimed to examine treatment outcomes among a sample of people with SSD who completed ≥ 4 intervention sessions in an 8-week MBCT program. We hypothesized that significant changes in psychosomatic distress, self-compassion, and alexithymia would be observed at 6-month follow-up following treatment inclusion. Second, this study further explored the mediating effects of self-compassion and alexithymia on MBCT outcomes in terms of psychosomatic distress by expanding on a previous process analysis. We also hypothesized that reduced psychosomatic distress would be mediated by improvements in self-compassion and alexithymia.

## Methods

### Study design and participants

**Figure 1:**
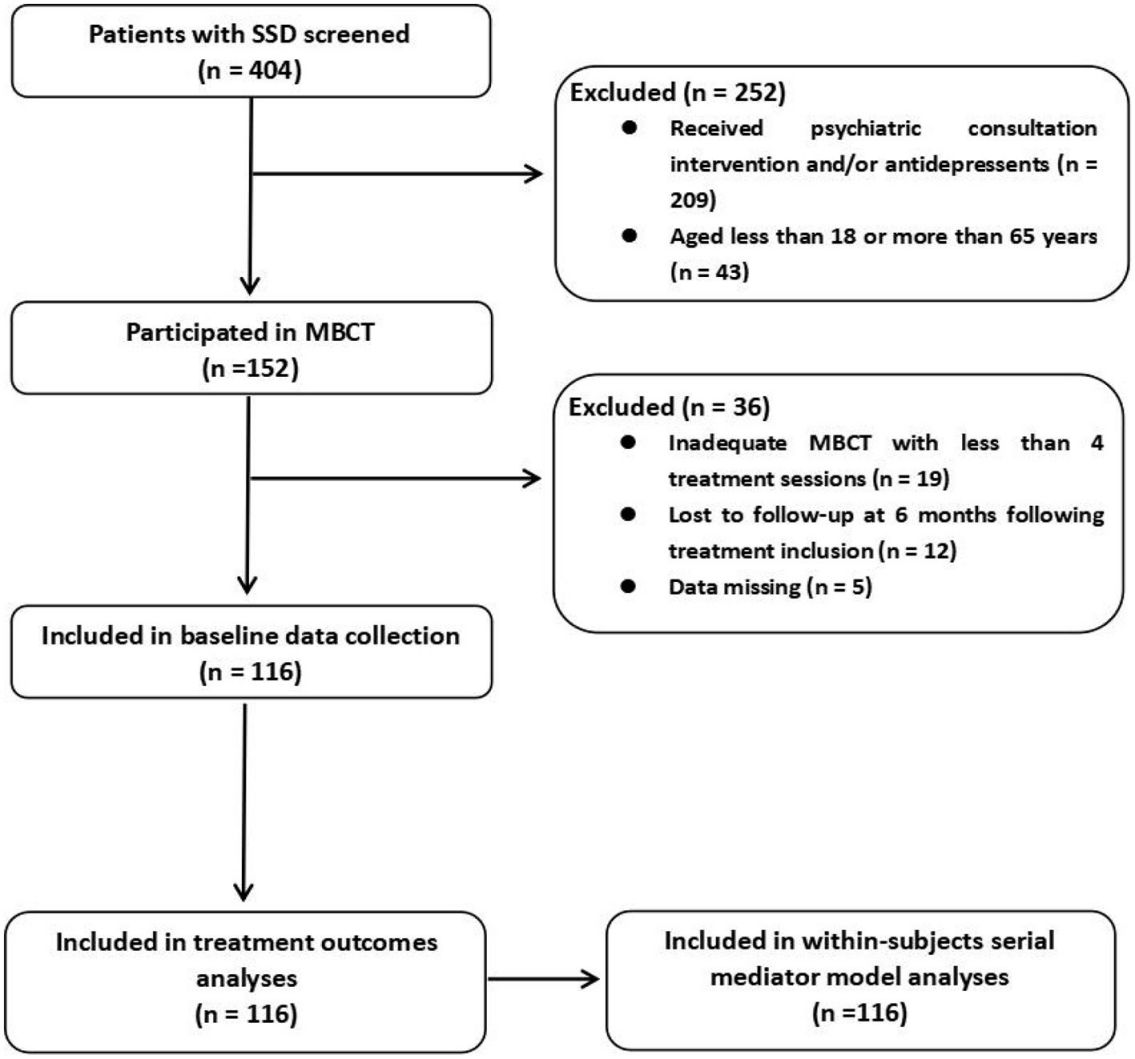
Study flowchart. SSD, somatic symptom disorder; MBCT, mindfulness-based cognitive therapy.

This was a retrospective cohort study. No ethical approval was required for this secondary analysis of existing data from previous medical records. Informed consent was obtained from all participants before treatment inclusion, and the study was approved by and conducted in accordance with the hospital's ethics review board. The eligibility criteria for the current study were patients who met the DSM-5 diagnostic criteria for SSD with more than 6 months' duration, who were aged between 18 and 65 years, and who underwent an 8-week MBCT program in Jining No.1 People's Hospital between July 2018 and December 2020. Over 400 patients with SSD were recruited through outpatient clinic screening and allocated to receive either a psychiatric consultation intervention or MBCT (Figure 1).

Participants were excluded from receiving the MBCT intervention if they had severe psychopathology (psychotic disorder, bipolar disorder, depressive disorder, substance-related disorders, severe personality disorders, etc.), a severe and unstable physical illness, cancer diagnosed within 5 years, a history of CBT, significant cognitive or visual impairment, or poor language skills that would hinder comprehension of the intervention or measurement. Patients who underwent fewer than 4 sessions of MBCT or for whom data on evaluated parameters were missing were also excluded from this study. Data and patient identification details were anonymized before analysis.

### Intervention

The MBCT intervention was conducted by one licensed mental health specialist (JS) with more than 2 years of experience in independently delivering MBCT education and training to ~100 patients. The MBCT program used in this study was based on the MBCT format for patients with depression (34). The Chinese MBCT program has been successfully applied in other studies (35, 36). We made minor modifications to render the MBCT training protocol suitable for the clinical setting of SSD. First, the cognitive components of MBCT included psychoeducation in respecting physical and mental boundaries in session 3 and identifying negative automatic thoughts in session 4. Second, to make it easier for patients to participate in the MBCT program, sessions were scheduled once a week or every other week for 8 consecutive weeks, with sessions lasting 90 min, in groups of 3–5 participants; the daily homework exercises guided by audio recordings after the weekly sessions were shortened to 30 min per day; and the content of the precourse introduction was integrated into session 1. Third, loving-kindness meditation was introduced in session 2 to cultivate kindness within participants and compassion for their somatic symptoms. Practices in the MBCT program mainly included guided body scan, mindful breathing, mindful walking, mindful awareness, loving-kindness meditation, and sitting meditation. Medical education on how to use medications properly also was added into the MBCT program. In each session, the therapist introduced the therapeutic modules and explained how to carry out the mindfulness exercises. Adequate participation in the MBCT program was defined as having attended at least 4 sessions, as in previous trials (37). The study data were collected upon admission to the MBCT program and at 6-month follow-up following treatment inclusion using self-report questionnaires or measures administered via face-to-face interviews.

### Measures

#### Outcome variables

The outcome measures were Brief Symptom Inventory-18 (BSI-18) score and the patient's Global Impression of Change (GIC). In this study, the Global Severity Index (GSI), derived from the BSI-18 data, was used for the mediation analysis.

#### Global impression of change

The GIC evaluates overall health status as perceived by patients on a 7-point Likert scale ranging from 1 (very much worse) to 7 (very much improved) relative to their status at treatment inclusion. Evidence has shown the validity of the GIC in assessing change in fibromyalgia, a diagnostic label overlapping substantially with SFD (38). A clinical improvement was defined as a GIC score of ≥ 5.

#### Brief symptom inventory-18

The severity of psychosomatic distress was assessed using the BSI-18, adapted from the original Symptom Checklist-90 Revised. The BSI-18 has 18 items scored on a 5-point Likert scale ranging from 0 (not at all) to 4 (extremely), and consists of three six-item subscales of somatization, anxiety, and depression, as well as a General Symptom Index (GSI) derived from the three subscales. The subscale scores and the GSI were calculated by summing scores. For the sample in this study, the BSI-18 had strong internal consistency and validity (Cronbach's α of 0.74 to 0.88 for each subscale and the global scale), similar to the values obtained in previous studies (39–41).

#### Mediators

The pre- and post-intervention data obtined using two process outcome measures, the Self-Compassion Scale (SCS) and the Toronto Alexithymia Scale (TAS), were used.

#### Self-compassion scale

The Chinese version of the SCS is a 26-item self-report measure that assesses three components of self-compassion, comprising three positive subscales (self-kindness, common humanity, and mindfulness) and three negative subscales (self-judgment, isolation, and over-identification). Items are scored on a 5-point Likert scale ranging from 1 (almost never) to 5 (almost always) (42). A high SCS score indicates a high level of self-compassion. Evidence has demonstrated the validity and reliability of the SCS when used in the form of the total score (43) and a positive or negative component summary (SCS-pos or SCS-neg) in a two-factor model (44). The total score is calculated by reverse-scoring the negative subscale items, calculating scores on the positive subscales, and then calculating the mean of all these subscales. To calculate scores for each subscale, SCS-pos and SCS-neg, a mean score is computed without reverse-scoring any of the items. In the current study, Cronbach's α was 0.93 for the total SCS, 0.85 for the SCS positive component summary, and 0.82 for the SCS negative component summary, suggesting a good degree of internal consistency.

#### Toronto alexithymia scale

Alexithymia was measured using the Chinese version of the 20-item TSA (TAS-20), which includes three subscales: difficulty in identifying feelings (DIF), difficulty in describing feelings (DDF), and externally oriented thinking (EOT). Items are scored on a 5-point Likert scale ranging from 1 (strongly disagree) to 5 (strongly agree), with a total score of ≥ 61 as the cut-off point for alexithymia (45, 46). Evidence has demonstrated the factorial validity and reliability of the TAS-20 (46, 47). In the present study, Cronbach's α was 0.80 for total TAS-20 score and 0.75, 0.72, and 0.62 for the subscales of DIF, DDF, and EOT, respectively.

### Data collection

All data, including demographics and evaluated variables, were obtained by reviewing electronic medical records. Demographic data included age, gender, illness duration, whether the patient was taking prescription antidepressants, marital status, education level, and employment status. Evaluated variables included clinical and process outcomes. The clinical outcome measure was the GIC score at 6-month follow-up following treatment inclusion (post-treatment follow-up). The process outcome measures were BSI, SCS, and TAS scores at treatment inclusion (pre-treatment) and post-treatment follow-up.

### Data analysis

Data analysis was conducted using SPSS (version 26.0; SPSS Inc.). The tested mediation model was estimated using MEMORE (version 2.1) for SPSS. Continuous data were tested for normality using the Shapiro–Wilk test, and if normally distributed, the data were expressed in the form mean ± standard deviation (SD) and tested using paired *t*-tests for intergroup comparisons. Changes in measures from pre-treatment to post-treatment follow-up were summarized with means (95% confidence intervals). To assess the differences in post-treatment follow-up measures between patients taking antidepressants and those not taking antidepressants, linear models were used with the GIC score at post-treatment follow-up as the outcome variable and the indicator of antidepressants as the independent variable, adjusting for other pre-treatment covariates. Cohen's *d* effect sizes were calculated by dividing the mean difference between pre-treatment and post-treatment follow-up by the standard deviation of the difference; Cohen's *d* values of 0.20 to 0.50, 0.50 to 0.80, and > 0.80 represent small, moderate, and large effects, respectively (48). Bivariate correlations were calculated to examine the relationships between changes in psychometric variables of the Brief Symptom Inventory General Symptom Index (BSI-GSI), SCS (SCS-pos, SCS-neg, and total score), and TAS (DIF, DDF, EOT, and total score); correlation coefficients (r) of < 0.10, 0.10 to 0.30, 0.30 to 0.50, and 0.50 to 1.00 were considered to represent weak, small, moderate, and strong correlations, respectively (48, 49). Multivariate linear regression analysis was performed to assess the potential mediation effects of changes in the SCS dimensions (SCS-pos and SCS-neg) and the TAS dimensions (DIF, DDF, and EOT) as predictors of the GIC score and change in the BSI-GSI with adjustment for demographics and the corresponding pretreatment score. A within-subjects serial multiple-mediator model was further used to assess the mediating effects of self-compassion and alexithymia on the BSI-GSI. Confidence intervals for parameter estimates were determined from 5,000 bootstrapped samples. The threshold for statistical significance was *p* < 0.05.

## Results

### Patient characteristics

One hundred fifty-two patients were enrolled in an 8-week MBCT program, among whom 19 subjects attended fewer than 4 treatment sessions, 12 subjects were lost to follow-up at 6 months following treatment inclusion, and measurement data were missing for five subjects. Thus, 116 participants were eligible for this cohort analysis, of whom 32 (27.6%) were taking prescribed antidepressants upon entering the program (Figure 1). The demographics and clinical characteristics of the 116 patients who met the analysis criteria are presented in Table 1. The clinical improvement rate following MBCT was 64.7% as indicated by GIC scores. No difference in clinical improvement was found between patients taking prescribed antidepressants and those who were not upon entering the program.

**Table 1:** Demographic and clinical characteristics (*N* = 116).

**Variable**	***n* (%)**
Age [years, M (SD)]	40.0 (9.5)
Female gender [*n* (%)]	89 (76.7)
Illness duration [years, M (SD)]	6.8 (6.4)
Taking prescription antidepressants at inclusion [*n* (%)]	32 (27.6)
**Marital status [*****n*** **(%)]**	
Married or living with a partner	107 (92.2)
Divorced/widowed	4 (3.4)
Single	5 (4.3)
**Education level [*****n*** **(%)]**	
Primary school or lower	30 (25.9)
Secondary school	55 (47.4)
University or above	31 (26.7)
**Employment status [*****n*** **(%)]**	
Employed/retired	60 (51.7)
Disability pension or flexible work	47 (40.5)
On sick leave	4 (3.4)
Unemployed	5 (4.3)
Clinical improvement with a GIC score of ≥ 5 [*n* (%)]	75 (64.7)

GIC, the patient's Global Impression of Change.

### Evaluation of psychometric outcomes

The psychometric outcomes from pre-treatment to post-treatment follow-up are shown in Table 2. Compared with the pre-treatment values, the post-treatment values of the total score and the dimensional scores on the BSI-18, the SCS, and the TAS decreased or increased significantly (all *p* values < 0.001).

**Table 2:** Changes in psychometric variables from pre-treatment (pre) to post-treatment follow-up (post).

**Variable**	**Pre [M (SD)]**	**Post [M (SD)]**	**Change [Δ, M (95% CI)]**	**Cohen's *d***	***p*-value**
**BSI-18**					
Somatization	8.09 (5.10)	6.33 (4.47)	−1.77 (−2.16 to −1.37)^a^	0.82	< 0.001
Depression	5.78 (3.96)	4.63 (3.20)	−1.16 (−1.51 to −0.80)^a^	0.61	< 0.001
Anxiety	7.52 (4.91)	6.23 (3.80)	−1.28 (−1.64 to −0.93)^a^	0.67	< 0.001
GSI	21.40 (9.57)	17.19 (7.86)	−4.21 (−4.85 to −3.56)^a^	1.20	< 0.001
**SCS**					
SCS-pos	3.34 (0.77)	3.53 (0.65)	0.20 (0.14–0.26)	0.60	< 0.001
SCS-neg	3.31 (0.60)	3.01 (0.48)	−0.30 (−0.36 to −0.24)^a^	0.95	< 0.001
SCS total score	2.52 (0.52)	2.76 (0.40)	0.25 (0.30–0.20)	1.01	< 0.001
**TAS**					
DIF	19.64 (5.72)	14.6 (4.96)	−5.03 (−5.54 to −4.53)^a^	1.83	< 0.001
DDF	15.98 (4.22)	13.62 (3.77)	−2.36 (−2.83 to −1.89)^a^	0.92	< 0.001
EOT	18.63 (6.20)	17.45 (4.83)	−1.18 (−1.61 to −0.75)^a^	0.51	< 0.001
TAS total score	54.25 (10.35)	45.67 (9.73)	−8.58 (−9.35 to −7.80)^a^	2.04	< 0.001

^a^Negative values for change indicate improvement. BIS, Brief Symptom Inventory; GSI, General Symptom Index; SCS, Self-Compassion Scale; SCS-pos, SCS positive summary; SCS-neg, SCS negative summary; TAS, Toronto Alexithymia Scale; DIF, Difficulty in Identifying Feelings; DDF, Difficulty in Describing Feelings; EOT, Externally Oriented Thinking; CI, confidence interval.

### Inter-correlations among changes in psychometric variables

The relationships among the changes in psychometric variables are presented in the correlation matrix in Table 3, indicating that most of the correlations were significant and in the expected direction.

**Table 3:** Inter-correlations between pre–post changes in psychometric outcomes.

**Variable**	**1**	**2**	**3**	**4**	**5**	**6**	**7**	**8**
1 ΔBSI-GSI	—							
2 ΔSCS-pos	−0.496^***^	—						
3 ΔSCS-neg	0.537^***^	−0.164	—					
4 ΔSCS total score	−0.677^***^	0.769^***^	−0.757^***^	—				
5 ΔTAS-DIF	0.508^***^	−0.187^*^	0.392^***^	−0.378^***^	—			
6 ΔTAS-DDF	0.050	0.035	0.049	−0.008	0.052	—		
7 ΔTAS-EOT	0.370^***^	−0.360^***^	0.344^***^	−0.462^***^	−0.124	−0.088	—	
8 ΔTAS total score	0.567^***^	−0.301^**^	0.477^***^	−0.508^***^	0.616^***^	0.597^***^	0.421^***^	—

^*^p < 0.05, ^**^p < 0.01, ^***^p < 0.001. BIS-GSI, Brief Symptom Inventory General Symptom Index; SCS, Self-Compassion Scale; SCS-pos, SCS positive summary; SCS-neg, SCS negative summary; TAS, Toronto Alexithymia Scale; DIF, Difficulty in Identifying Feelings; DDF, Difficulty in Describing Feelings; EOT, Externally Oriented Thinking.

### Potential mediating effects on outcome of changes in self-compassion and alexithymia

Standard linear regression was conducted to examine the potential mediating effects of improvement in self-compassion and alexithymia on the clinical outcome and psychosomatic distress. As shown in Table 4, decreases in SCS-neg, DIF, and EOT values and increases in SCS-pos values were each associated with GIC score and with a greater reduction in BSI-GSI value when adjusting for pretreatment covariates.

**Table 4:** Interactive effects of changes in SCS dimensions and TAS dimensions as potential mediators in predicting GIC and change in BSI-GSI with adjustment for pretreatment covariates.

**Variable**	***B***	**β**	***t***	***p***
**GIC as the clinical outcome**				
ΔSCS-pos	1.100	0.317	5.370	< 0.001
ΔSCS-neg	−1.001	−0.282	−4.395	< 0.001
ΔTAS -DIF	−0.162	−0.392	−6.255	< 0.001
ΔTAS -EOT	−0.146	−0.301	−4.674	< 0.001
Δ**BSI-GSI indicating reduction in psychosomatic distress**				
ΔSCS-pos	−3.257	−0.305	−4.377	< 0.001
ΔSCS-neg	2.922	0.268	3.535	0.001
ΔTAS -DIF	0.475	0.373	5.040	< 0.001
ΔTAS -EOT	0.320	0.214	2.816	0.006

GIC, Global Impression of Change; BSI-GSI, Brief Symptom Inventory General Symptom Index; SCS, Self-Compassion Scale; SCS-pos, Self-Compassion Scale positive summary; SCS-neg, Self-Compassion Scale negative summary; TAS, Toronto Alexithymia Scale; DIF, Difficulty in Identifying Feelings; DDF, Difficulty in Describing Feelings; EOT, Externally Oriented Thinking.

### Indirect effect of treatment via changes in self-compassion and alexithymia as mediators

**Figure 2:**
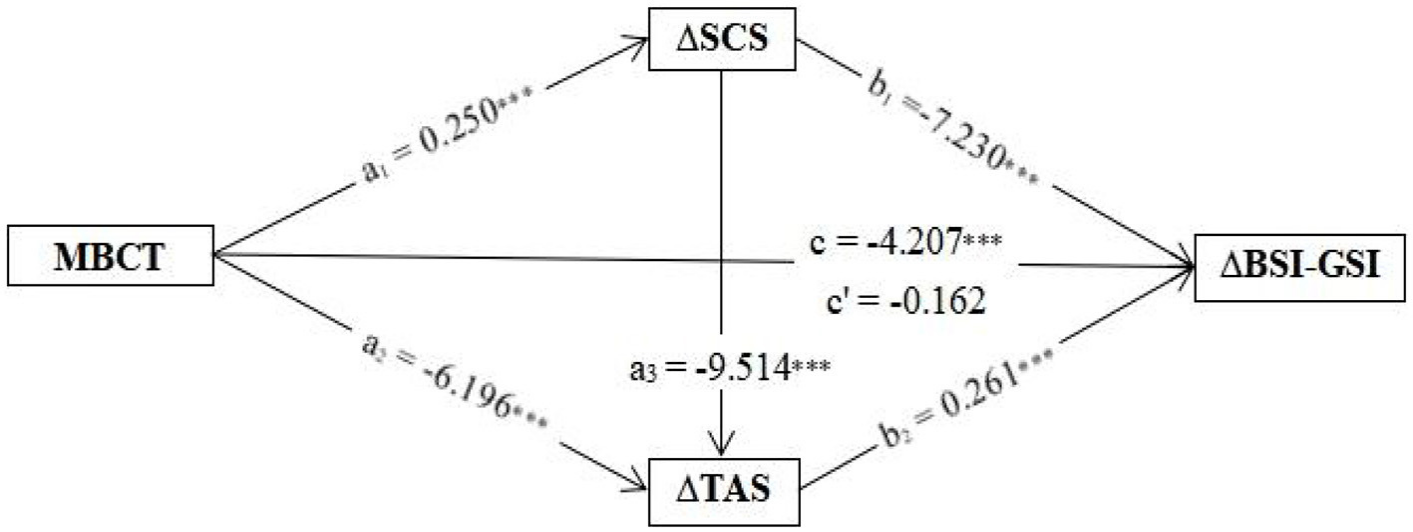
The path analysis results of a serial multiple-mediator model for changes in self-compassion and alexithymia predicting change in BSI-GSI following MBCT. The results indicate significant indirect effects mediating the outcome for self-compassion and alexithymia, and the serial effect of change in SCS score influencing the subsequent change in TAS score. Coefficients represent beta values for the displayed parameter. ***p < 0.001. MBCT, mindfulness-based cognitive therapy; BSI-GSI, Brief Symptom Inventory General Symptom Index; SCS, Self-Compassion Scale; SCS-pos, Self-Compassion Scale positive summary; SCS-neg, Self-Compassion Scale negative summary; TAS, Toronto Alexithymia Scale; DIF, Difficulty in Identifying Feelings; DDF, Difficulty in Describing Feelings; EOT, Externally Oriented Thinking.

We further sought to explore how concurrent changes in SCS values and TAS values as mediators impacted perceived psychosomatic distress, as indicated by BSI-GSI scores, using a serial multiple-mediator model. In serial mediation, each mediator is assumed to causally influence subsequent mediators: for instance, an increase in self-compassion is predicted to cause a decrease in alexithymia. Given that these are causal inferences, the estimated effects are unidirectional (MBCT→ΔSCS→ΔTAS→ΔBSI-GSI). The present serial mediation model revealed that changes in both SCS values and TAS values had significant indirect effects on the pre–post change in BSI-GSI value [ΔSCS β = −1.810 (a${\;}_{1}^{*}$b_1_), bootstrap SE = 0.341, 95% bootstrap CI (−2.488, −1.160); ΔTAS β = −1.615 (a${\;}_{2}^{*}$b_2_), bootstrap SE = 0.391, bootstrap 95% CI (−2.413, −0.896); ΔSCS→ΔTAS β = −0.621 (a${\;}_{1}^{*}$a${\;}_{3}^{*}$b_2_), bootstrap SE = 0.183, bootstrap CI (−1.032, −0.315)], as shown in Figure 2; see Supplementary Table S1 for the full model [*F*_(4,111)_ = 31.464, *p* < 0.001, *R*^2^ = 0.531, MSE = 5.924].

**Figure 3:**
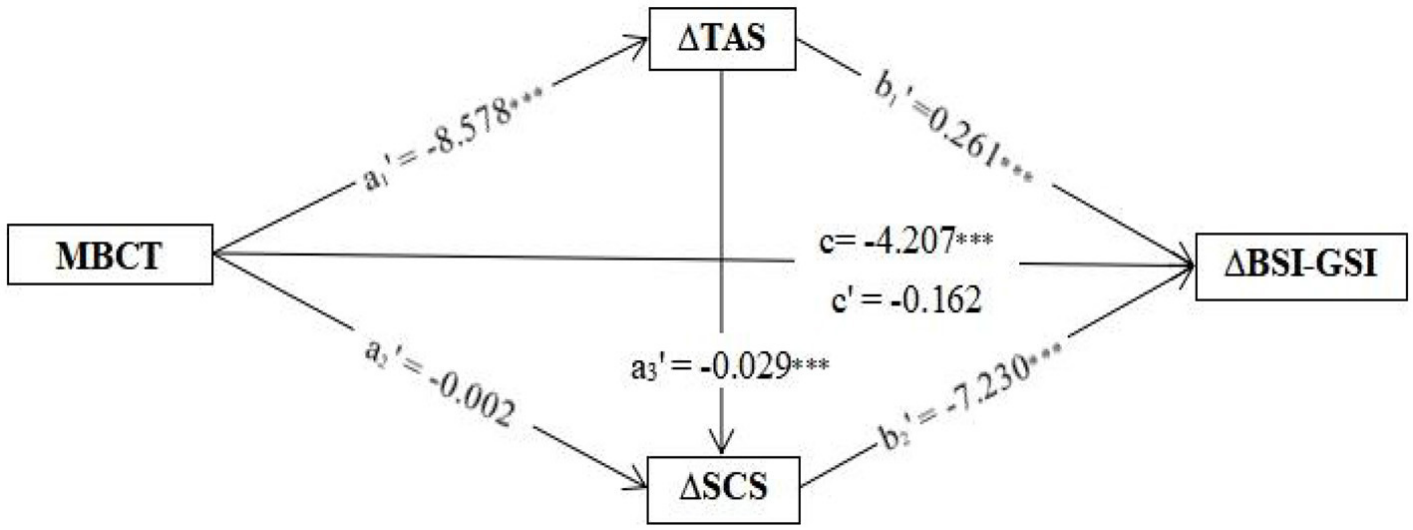
The path results of the reverse serial mediation effect (MBCT→ΔTAS→ΔSCS→ΔBSI-GSI) in a *post hoc* mediation analysis. Coefficients represent beta values for the displayed parameter. ***p < 0.001. MBCT, mindfulness-based cognitive therapy; BSI-GSI, Brief Symptom Inventory General Symptom Index; SCS, Self-Compassion Scale; SCS-pos, Self-Compassion Scale positive summary; SCS-neg, Self-Compassion Scale negative summary; TAS, Toronto Alexithymia Scale; DIF, Difficulty in Identifying Feelings; DDF, Difficulty in Describing Feelings; EOT, Externally Oriented Thinking.

In contrast, *post-hoc* analysis with a reverse sequence (MBCT→ΔTAS→ΔSCS→ΔBSI-GSI) revealed that only the change in TAS value still had a significant indirect effect on pre–post change in BSI-GSI value [ΔTAS β = −2.235 (a_1_'^*^b_1_'), bootstrap SE = 0.516, bootstrap 95% CI (−3.305, −1.270); ΔSCS β = 0.013 (a${'}_{2}^{*}$b_2_'), bootstrap SE = 0.323, 95% bootstrap CI (−0.600, 0.682); ΔTAS→ΔSCS β = −1.823 (a_1_'^*^a_3_'^*^b_2_'), bootstrap SE = 0.439, bootstrap CI (−2.770, −1.047)], as shown in Figure 3; see Supplementary Table S1 for the full model [*F*_(4,111)_ = 31.464, *p* < 0.001, *R*^2^ = 0.531, MSE = 5.924].

## Discussion

To the best of our knowledge, this might be the first study to explore improvements in self-compassion and alexithymia following an MBCT intervention and their serial mediating effects on psychosomatic distress. The main finding from the present study showed that a decrease in psychosomatic distress for SSD patients was associated with greater improvements in self-compassion and alexithymia following MBCT intervention. Mediation analyses in this study further showed that relief of psychosomatic distress following MBCT occurred directly via an increase in self-compassion (MBCT→ΔSCS→ΔBSI-GSI β = −1.810) and a decrease in alexithymia (MBCT→ΔTAS→ΔBSI-GSI β = −1.615), and indirectly (partially) through subsequent reduction in alexithymia following the improvement in self-compassion (MBCT→ΔSCS→ΔTAS→ΔBSI-GSI β = −0.621).

As the patients' GIC scores at 6-month follow-up indicated, the MBCT intervention was significantly effective in relieving somatic symptom distress for SSD patients, which is congruent with previous findings in the literature (17, 50, 51). Mindfulness practice, as a core component of MBIs (e.g., MBCT or MBSR), is considered to one way of developing the implicit skill of promoting self-compassion in the context of overall wellbeing (21). There is also evidence for the efficacy of MBCT in improving self-compassion and for mediating effects of self-compassion on treatment outcomes (15, 19, 21). The treatment process analyses in this study yielded a similar result to a previous study (25): namely, improvement in self-compassion accounted for reductions in physical symptoms and psychological strains at post-treatment follow-up to a certain extent. Evidence has shown that mindfulness and alexithymia are negatively related constructs (high alexithymia is associated with low mindfulness) (52). Recent systematic reviews have concluded that MBIs might be an effective means of reducing alexithymia or increasing emotional clarity (31, 32). The treatment process analyses in this study also identified the role of reduction in alexithymia in accounting for significant portions of the variance in change in perceived psychosomatic distress, especially DIF and EOT. However, in a previous study (53), higher EOT was identified as a resilience factor in the face of life adversity. Additionally, Butler et al. found that the MBSR program was equivalent to a CBT program in terms of improvements in emotional clarity in patients with social anxiety disorder and did not observe a moderating effect of emotional clarity on the outcome of treatment for social anxiety (33).

Moreover, our analyses demonstrated that both self-compassion and alexithymia mediated the relationship between MBCT and changes in psychosomatic distress. The conclusions might not be surprising, given the robust literature identifying associations of self-compassion and alexithymia with psychosomatic symptoms and diminished life quality among SSD patients (54–57). Although studies have suggested that self-compassion is directly associated with the treatment outcomes of MBIs (19), no study has detailed the specific processes that occur through self-compassion and its association with alexithymia. Notably, our *post-hoc* serial mediation analysis in a reverse sequence (MBCT→ΔTAS→ΔSCS→ΔBSI-GSI) also achieved statistical significance in terms of indirect mediating effects on psychosomatic distress [β = −1.823, bootstrap SE = 0.440, 95% bootstrap CI (−2.7963, −1.072)], but it did not demonstrate that a change in self-compassion exerted a significant indirect effect on psychosomatic distress [β = 0.013, bootstrap SE = 0.3421, 95% bootstrap CI (−0.577, 0.682)]. These results showed that MBCT could potentially result in a reduction in alexithymia, and the MBCT-related reduction in alexithymia subsequently led to an increase in self-compassion; these findings are partially supported by the finding that fear of self-compassion and fear of happiness fully mediate the effects of alexithymia upon depression in a previous study with a depressive sample (58). A recent study involving a depressive sample has further identified self-compassion as an outcome of psychotherapy and fear of compassion as a putative mechanism by which depressive symptoms are alleviated (59).

### Study limitations

Several limitations should be considered when interpreting the results of our analysis. First, this was a single-center retrospective study with a small sample size. There was some degree of selection bias. Second, no participant was recruited into a control group in this study, which limits the ability to compare the outcomes with natural changes in psychosomatic distress, self-compassion, and alexithymia over time. Third, this study was only designed to explore the impact of implicit self-compassion on alexithymia. The MBCT program is a multi-component program. Other active components that were not assessed in this study, such as mindfulness, acceptance, emotions, cognition, and behaviors, need to be tested for association with alexithymia. Previous research has suggested that increased mindfulness is associated with a decrease in DIF (60). Additionally, many participants were also treated with antidepressants, and the types of antidepressants varied. Changes in self-compassion and alexithymia following the MBCT intervention might be enhanced by these concomitant medications. Fourth, alexithymia as a personality trait is transdiagnostic and linked to a range of psychiatric morbidities, such as depression, panic disorder, eating disorders, chronic pain disorders, substance abuse, and suicide (53). The findings from a small sample of SSD patients were limited in their ability to guide clinical practice in terms of generalizability.

## Conclusion

Both alleviation of alexithymia and improvement in self-compassion following an MBCT program are partially responsible for reducing psychosomatic distress in SSD patients. The results from the mediation analysis with the sequence of MBCT→ΔTAS→ΔSCS→ΔBSI-GSI suggested that improvement in self-compassion might be an outcome of an MBCT-related reduction in alexithymia. Future studies are required to support this hypothesis and develop sensitive techniques for addressing alexithymia in clinical practice.

## Data availability statement

The original contributions presented in the study are included in the article/Supplementary material, further inquiries can be directed to the corresponding author.

## Ethics statement

Ethical approval was not required for the study involving humans in accordance with the local legislation and institutional requirements. Written informed consent to participate in this study was not required from the participants or the participants' legal guardians/next of kin in accordance with the national legislation and the institutional requirements.

## Author contributions

LX: Data curation, Formal analysis, Investigation, Writing – original draft, Writing – review & editing. JS: Conceptualization, Formal analysis, Funding acquisition, Investigation, Methodology, Supervision, Writing – review & editing. CL: Conceptualization, Data curation, Writing – original draft, Writing – review & editing.
