# Supplementary material for: Addressing psychosomatic symptom distress with mindfulness-based cognitive therapy in somatic symptom disorder: mediating effects of self-compassion and alexithymia
Source: Front Psychiatry. 2024 Feb 7;15:1289872. doi: 10.3389/fpsyt.2024.1289872 (PMC10879322; doi:10.3389/fpsyt.2024.1289872)
Supplement: Supplementary file 1 [file Table_1.docx]

| **SUPPLEMENTARY TABLE 1:** Direct and indirect effects on change in Brief Symptom Inventory estimated within‐participant serial mediation model | | | | | |
| --- | --- | --- | --- | --- | --- |
| **Direct effect (path)** | ***β*** | **SE** | ***t*** | ***p*** | **CI** |
| MBCT→ΔBSI-GSI (c) | -4.207 | 0.324 | -12.972 | < 0.001 | -4.849 - -3.565 |
| MBCT→ΔBSI-GSI (c') | -0.162 | 0.526 | -0.308 | 0.759 | -1.205 - 0.880 |
| MBCT→ΔSCS (a_1_) | 0.250 | 0.023 | 10.922 | < 0.001 | 0.205 - 0.296 |
| MBCT→ΔTAS (a_2_) | -6.196 | 0.516 | -12.015 | < 0.001 | -7.218 - -5.175 |
| ΔSCS→ΔTAS (a_3_) | -9.514 | 1.559 | -6.101 | < 0.001 | -12.603 - -6.424 |
| MBCT→ΔTAS (a_1_') | -8.578 | 0.390 | -21.976 | < 0.001 | -9.351 - -7.804 |
| MBCT→ΔSCS (a_2_') | -0.002 | 0.046 | -0.040 | 0.968 | -0.093 - 0.089 |
| ΔTAS→ΔSCS (a_3_') | -0.029 | 0.005 | -6.118 | < 0.001 | -0.039 - -0.020 |
| ΔSCS→ΔBSI-GSI (b_1_/b_2_') | -7.230 | 1.206 | -5.996 | < 0.001 | -9.619 - -4.840 |
| ΔTAS→ΔBSI-GSI (b_2_/b_1_') | 0.261 | 0.063 | 4.109 | < 0.001 | 0.135 - 0.386 |
| **Indirect effect (path)** | ***β*** | **Bootstrap SE** | | **Bootstrap CI** | |
| MBCT→ΔSCS→ΔBSI-GSI (a_1_*b_1_) | -1.810 | 0.339 | | -2.469 - -1.136 | |
| MBCT→ΔTAS→ΔBSI-GSI (a_2_*b_2_) | -1.615 | 0.381 | | -2.376 - -0.907 | |
| MBCT→ΔSCS→ΔTAS→ΔBSI-GSI (a_1_*a_3_*b_2_) | -0.621 | 0.178 | | -1.013 - -0.320 | |
| MBCT→ΔTAS→ΔBSI-GSI (a_1_'*b_1_') | -2.235 | 0.516 | | -3.305 - -1.270 | |
| MBCT→ΔSCS→ΔBSI-GSI (a'_2_*b_2_') | 0.013 | 0.323 | | -0.600 - 0.682 | |
| MBCT→ΔTAS→ΔSCS→ΔBSI-GSI (a_1_'*a_3_'*b_2_') | -1.823 | 0.439 | | -2.770 - -1.047 | |
| MBCT, Mindfulness-based cognitive therapy; BSI-GSI, Brief Symptom Inventory General Symptom Index; SCS, Self-Compassion Scale; TAS, Toronto Alexithymia Scale. | | | | | |
